# Editorial

**DOI:** 10.1080/11287462.2017.1302184

**Published:** 2017-03-17

**Authors:** Patricia Kingori, Michael Parker

We are delighted to have become the new editors of *Global Bioethics*. In the coming years, we aim – with your help – to make this the most exciting and innovative place to publish and to read about the ethical and social aspects of global health research and policy. One of our most important ambitions is that the Journal should include voices from, and be read in, both high- and low-income settings. For that reason, we are particularly pleased that the beginning of our editorship coincides with *Global Bioethics* becoming an Open Access Journal and it is also for thisreason that we have established a new “field notes” section – of which more below.

In recent years, there has been a dramatic increase in the scale and scope of global health research. This has been accompanied by academic and policy literature highlighting the importance of identifying and addressing the ethical and social issues presented by such research. It is of pressing international importance that global health research is conducted successfully to high ethical standards capable of commanding public trust in diverse settings. Constructive, critical and inclusive multidisciplinary debate of the issues is a key part of this. There is, however, a significant absence of forums where scholars from diverse global contexts can publish alongside each other. Furthermore, the framing of discussions in terms of global North/South, developing or developed, in those forums that do exist, has tended to tie the discussion of ethical issues to geographical location rather than, often complex and international, context and this has limited our understanding of bioethics as a global phenomenon. It is our view that a contextual rather than geographically reductive approach to bioethics is called for. This is illustrated, for example, by the recent economic crises faced in many European countries and North America at the same time that many countries in the global South have witnessed unprecedented economic growth.

*Global Bioethics* provides a broad open access international multidisciplinary forum for research on the ethical aspects of global health research, practice and policy. It brings together scholarship from around the world covering a variety of socio-economic and cultural contexts and ethical positions in order to reflect and promote new critically engaged ways of thinking about contemporary topics in global bioethics.

The Journal aims to be close to practice and to engage with ethical issues faced by real-world actors in real-world settings. One implication of this is that it places particular emphasis on research bringing together empirical research with rigorous ethical analysis, and which pays attention to political and social structures alongside macro-level analysis of global bioethical issues. Theoretical and methodological papers in global bioethics are also welcomed.

*Global Bioethics* will publish research and review papers, editorials and commentaries, and analysis and correspondence articles. We will welcome research on matters related to global bioethics from a range of genres, and in all study phases and designs. One of our keys aims is to reflect live issues through the Journal’s “field notes” section which comprises shorter observations and initial findings from “the field”, that is, from day-to-day practice. Recognising the interdisciplinary nature of research on bioethical issues, we will publish studies using social science methods and approaches, and also philosophical papers addressing bioethical issues. Our aim is to be a forum where scholars from around the world, at different levels of seniority and employing different disciplines and methodologies can come together.

In line with these aims *Global Bioethics* is now an Open Access Journal which publishes thought-provoking and insightful empirical and normative research articles, field notes and commentary articles. All submitted manuscripts are subject to initial appraisal by Editors and Editorial Board Members, and then, where appropriate, to a rigorous, fast and anonymous double-blind peer review by independent expert referees. All submissions should be made via http://www.edmgr.com/rgbe/default.aspx.

